# Receptor–ligand pair typing and prognostic risk model of response or resistance to immune checkpoint inhibitors in lung adenocarcinoma

**DOI:** 10.3389/fonc.2023.1170942

**Published:** 2023-04-19

**Authors:** Shengqiang Mao, Lingyan Zeng, Ying Yang, Zhiqiang Liu, Li Zhang

**Affiliations:** Department of Respiratory and Critical Care Medicine, Frontiers Science Center for Disease-related Molecular Network, Center of Precision Medicine, Precision Medicine Key Laboratory of Sichuan Province, West China Hospital, Sichuan University, Chengdu, Sichuan, China

**Keywords:** lung adenocarcinoma, immunotherapy, cell-cell interactions, ligand-receptor interactions, single-cell RNA sequencing

## Abstract

**Introduction:**

Currently, programmed cell death-1 (PD-1)-targeted treatment is ineffective for a sizable minority of patients, and drug resistance still cannot be overcome.

**Methods:**

To explore the mechanisms of immunotherapy and identify new therapeutic opportunities in lung adenocarcinoma (LUAD), data from patients who did and did not respond to the anti-PD-1 treatment were evaluated using single-cell RNA sequencing, and bulk RNA sequencing were collected.

**Results:**

We investigated the gene expression that respond or not respond to immunotherapy in diverse cell types and revealed transcriptional characteristics at the single-cell level. To ultimately explore the molecular response or resistance to anti-PD-1 therapy, cell-cell interactions were carried out to identify the different LRIs (ligand-receptor interactions) between untreated patients vs. no-responders, untreated patients vs. responders, and responders vs. non-responders. Next, two molecular subgroups were proposed based on 73 LRI genes, and subtype 1 had a poor survival status and was likely to be the immunosuppressive tumor subtype. Furthermore, based on the LASSO Cox regression analysis results, we found that *TNFSF13, AXL, KLRK1, FAS, PROS1*, and *CDH1* can be distinct prognostic biomarkers, immune infiltration levels, and responses to immunotherapy in LUAD.

**Discussion:**

Altogether, the effects of immunotherapy were connected to LRIs scores, indicating that potential medications targeting these LRIs could contribute to the clinical benefit of immunotherapy. Our integrative omics analysis revealed the mechanisms underlying the anti-PD-1 therapy response and offered abundant clues for potential strategies to improve precise diagnosis and immunotherapy.

## Introduction

Lung cancer has been the leading cause of cancer death worldwide, accounting for 22.7% of malignant tumor classifications (1). Lung adenocarcinoma (LUAD) is the major histological subtype of non-small cell lung cancer (NSCLC) and accounts for more than 40% of all lung cancers (2). Therapeutic options for LUAD include surgery, radiation, chemotherapy, targeted therapy, immunotherapy, or a combination of these treatments that have achieved remarkable success in treatment (3). Nevertheless, there is still approximately 30% of LUAD cases lack available targeted therapeutic options (4, 5). EGFR-tyrosine kinase inhibitors (EGFR-TKIs) and immunotherapy have brought unprecedented survival benefits to LUAD patients, however acquired EGFR-TKI resistance mechanism and immunotherapy resistance also have become the new problems in the treatment (6, 7). The current treatment focus on overcoming various resistance mechanisms, precision therapy based on new therapeutic targets and new therapy is the key to improving the prognosis of LUAD (8, 9).

High-throughput sequencing technology provides convenience for the identification of new therapeutic targets. Recently, single-cell RNA sequencing (scRNA-Seq) has shown promise of unraveling the biology of LUAD at an unprecedented level of resolution. Several studies have explored LUAD at the single-cell level, focused on finding potential targets for early diagnosis and treatment, and described the significance of cellular interactions in the tumor microenvironment (10, 11). LRIs, as essential components of cell–cell communication, play a vital role mediating cellular communication and signal transduction. Immunotherapies that target ligand-receptor interactions (LRIs) have recently made significant strides in the treatment of various tumors, such as colorectal cancer (CRC) and bladder urothelial carcinoma (BLCA) (12, 13). In contrast, the pattern of LRIs in LUAD and their effect on tumor microenvironment and clinical value are still unclear (14, 15). The scRNA-seq has been used to study cell-cell interaction networks in the TME, revealing breast cancer anti-PD-L1 immunotherapy combined with chemotherapy alterations of molecular characterization in the TME (16, 17). Therefore, we envision that cell-cell interaction analyses based on scRNA-seq can contribute to determining critical cell-cell interactions involved in response or resistance to anti-PD-1 therapy.

In this study, we integrated bulk mRNA-seq, scRNA-seq, and genomics data collected from LUAD patients. mainly explored the mechanisms of response or resistance to anti-PD-1 therapy from untreated patients and several immunotherapy datasets. Based on pivotal LRIs, the new molecular characteristics and mapping of the immune cell infiltration landscape are developed. Several crucial LRIs play critical roles in immunotherapy and increase survival after anti-PD-1 therapy. Overall, our findings provide in-depth insight into the processes underlying the crucial cell-cell interactions that affect the effectiveness of anti-PD-1 treatment.

## Materials and methods

### Sample collection

Fresh tumor tissues were collected from patients with lung cancer at West China Hospital (WCH). All patients signed informed written consent. Clinical characteristics, including age, sex, smoking status, pathological subtype, and stage, were recorded at recruitment and are listed in **Supplementary Table S1**.

### Tissue dissociation and single-cell suspension preparation

Freshly obtained resected tissues were sliced into smaller pieces on ice with collagenase I/IV in HBSS and incubated for 30 minutes at 37°C with manual shaking every 5 minutes. Following the process, the tissues were washed with Hank’s balanced salt solution (HBSS) and cut into smaller pieces. The cells that were still in solution underwent a five-minute 500 g centrifugation. The supernatant was removed, the blasted cells were dispersed in red blood lysis solution, rinsed with HBSS, and then reconstituted in sorting buffer (0.04% BSA + PBS). After dead cells were cleared by flow cytometry, cell suspensions were promptly prepared for single-cell RNA-seq.

### Library preparation and sequencing

According to the manufacturer’s instructions, single cells were created using the Chromium Single Cell 3′ Gel Bead, Chip and Library Kits v2 (10X Genomics), total cells added to each channel ranged from 6000 to 10000 cells. Following cell lysis and barcoded reverse transcription of RNA, followed by amplification, shearing, and attachment of the 5′ adaptor and sample index. At Chengdu’s West China Hospital, libraries were sequenced using the Illumina NovaSeq 6000 platform.

### scRNA-seq data clustering, dimension reduction and cell annotation

The R package “Seruat” was used for the following single cell analysis. First, the data were integrated to use the “harmony” algorithm and normalized through the log-normalization function, and the top 2000 highly variable genes were identified through the “FindVariableFeatures” function. Next, all genes were scaled using the “ScaleData” function, and the “RunPCA” function was used to reduce the dimension of PCA for the highly variable genes. We chose dims=20 and clustered the cells through the “FindNeighbors” and “FindClusters” functions (resolution=0.5). UMAP is a method of data dimensionality reduction that assumes that the available data samples are uniformly distributed in the topological space, and these limited data samples can be approximated and mapped to a low-dimensional space.

The “FindAllMarkers” function was used to screen the marker genes of 13 subgroups with log2FC=0.25 and min.pct=0.25 (the expression ratio of the least differential genes). Finally, we used the corrected p < 0.01 to screen the marker gene. Cell type definitions were obtained from previous studies and manually annotated according to marker genes.

### Copy number alteration (CNA)

CNAs of each cell type were estimated by sorting the analyzed genes on chromosomal location and applying a moving average to the relative expression values, with a sliding window of 100 genes within each chromosome by inferCNV (18).

### Cell−cell communication analysis

Cell−cell communication analysis was performed using the “CellPhoneDB” function, significant mean and significance of cell communication based on cell interactions and normalized cell matrix by P test calculated. LR-pairs were obtained for each cell pair with nominal p < 0.05. Next, we conducted a deeper analysis of cell−cell interactions by linking ligand expression on one cell type to some target genes of interest expressing another cell type using NicheNet.

### Comparison of cell−cell interactions between untreated patients, responders and nonresponders

To identify ligand−receptor pairs with significant differences between pretreatment responders and nonresponders, we used the Mann−Whitney U test to compare the interaction scores of each ligand−receptor pair in all cell pairs between untreated patients and responders. Ligand−receptor pairs with an adjusted p value <0.05 were preserved. For visualization, the ratio of untreated patients/nonresponder interaction scores was calculated as follows:

Ratio (untreated patients to nonresponders) = Interaction frequency (untreated patient)/Interaction frequency (nonresponders).

If the ratio (untreated patient/nonresponders) >1, a higher level of interaction existed in responders than in nonresponders (indicated with a red color). If the ratio (untreated patient/nonresponders) <1, a lower level of interaction existed in responders than in nonresponders (indicated with a blue color).

### Gene set enrichment analysis and functional annotation

Gene Ontology (GO) and Kyoto Encyclopedia of Genes and Genomes (KEGG) analyses of DEGs were performed using the R package “ClusterProfiler”. Cellular component, biological processes, and molecular functions were all included in the GO enrichment analysis. Gene set enrichment analysis (GSEA) was used to examine the pathways connected to various molecular subgroups using all potential gene sets in the Molecular Signature Database.

To assess whether a gene set is enriched in a particular cell subpopulation, single cells were enriched for gene function using the “irGSEA” method. Each cell was scored separately by multiple gene set enrichment methods, and multiple gene set enrichment score matrices were generated. “irGSEA scores” were generated using preset gene sets collected from the “MSigDB” for cancer hallmark and signaling route signatures.

### Gene expression data, somatic mutation data and clinical information

**Figure 1:**
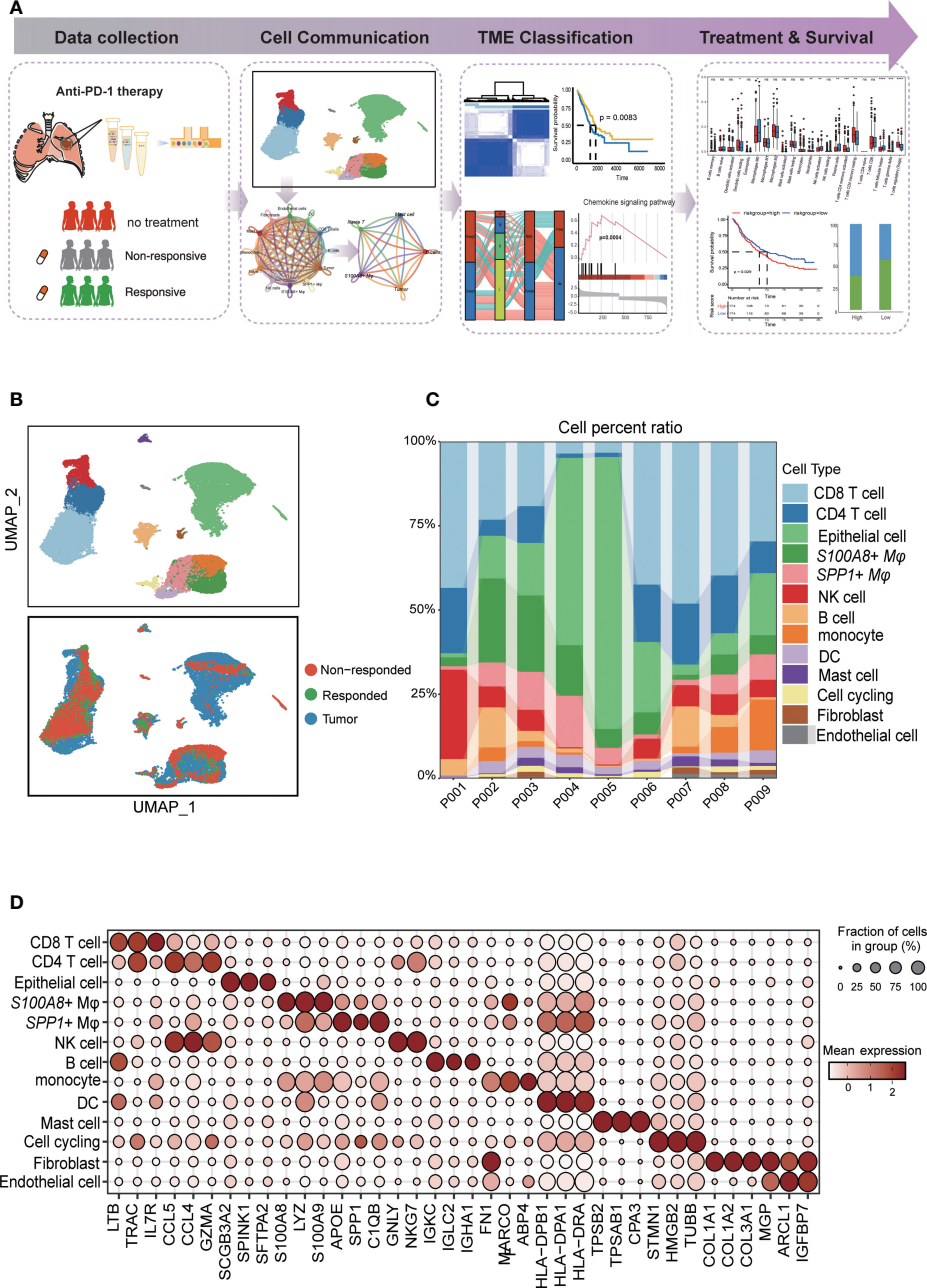
Diverse cell types in LUAD were delineated by single-cell RNA-seq analysis. **(A)** Schematic of tissue dissociation, cell isolation, sequencing, and downstream bioinformatics analysis. **(B)** UMAP plots of the major LUAD cell populations from untreated patients and responders and nonresponders after PD-L1 treatment. Each point depicts a single cell, colored according to cell type (top). The distribution of cells from three groups is divided by treatment or response (bottom). **(C)** Stacked bar plots showing the frequencies of 13 cell types in seven samples. The chart shows the number and percentage of each cell type. **(D)** Dot plots showing the single-cell expression pattern of cell-type-specific top 3 gene markers in each cell type.

For the training cohort, mRNA expression profiles, somatic mutation data and related clinical data of LUAD were accessed from the TCGA database *via* UCSC Xena (https://xena.ucsc.edu/). The somatic mutation data were analyzed *via* the “maftools” R package (version 2.2.10). The TMB scores were obtained by calculating the total number of mutations/exon length (38 Mb). The mRNA expression data and related clinical parameters used for validation cohorts were accessed from the GEO database (http://www.ncbi.nlm.nih.gov/geo/), including the GSE146100 dataset and GSE126045. Moreover, we performed log2 transformations for all mRNA expression data. Samples with a survival time < 30 days were excluded from this study. Finally, the flowchart of this research is shown in **Figure 1A**.

### LRI-related gene classifications of LUAD patients in the TCGA cohorts

The “ConsensusClusterPlus” R package was applied to explore the molecular classification of TCGA LUAD cohorts based on the expression pattern. For unsupervised consensus clustering based on k-means machine learning, 80% of the data were iterations of the 1,000-iter clustering algorithm. The prognosis of patients between the two subgroups was then assessed using the Kaplan−Meier survival analysis. To further investigate the relationships between the LRI genes and the clinical characteristics, we carried out comparisons of the histopathological features between the two subgroups.

### Cell-type and immune infiltration assessment between two subgroups

To precisely estimate the proportions of cell types in tissues, cell type deconvolution by the algorithm uses the input matrix of the gene expression dataset. Here, we compared variations among diverse immune cells in two groups using “CIBERSORT” algorithm. To investigate the relationship between the risk score and invading immune cells, Spearman correlation analysis was used, the correlation analysis results and variations in immune cell abundance were displayed using the ggplot2” R package.

### Establishment of the risk model

The R package “glmnet” was used to perform least absolute shrinkage and selection operator (Lasso) Cox regression and predict the prognosis for patients based on LRIs. First, each independent variable’s change trajectory was examined. Cross validation is used to select the tuning parameter. The cox.ph function in the R package “survival” was used to create the Cox proportional hazards model, which was then used to evaluate the consistency and variability of the estimates generated by the Lasso Cox regression model. Based on the median score, patients were separated into high- and low-expression groups, survival analysis was performed between two groups.

### Survival analysis and immunotherapeutic evaluation

Survival analysis was performed by the R package survival. The hazard ratio (HR) was calculated by the Cox proportional hazards model, the 95% CI was reported, and the Kaplan–Meier survival curve was modeled by the “survfit” function. The “maxstat.test” function of the R package “maxstat”, in which all potential cutting points were repeatedly tested to find the maximum rank statistic, was used to perform dichotomy of cell population infiltration or gene expression and then divide the patients into two groups according to the selected maximum logarithm statistics. The two-sided long-rank test was used to compare Kaplan–Meier survival curves. The comparison of the percentage of patients who responded to ICB treatment between different groups was determined by the Chi-Squared test.

### Statistical analysis

The R programming language was used for all statistical studies (version 4.2.0). For the correlation analysis, Spearman’s correlation was used. To examine the differences between these two risk groups, the Wilcoxon test was applied. Statistical significance was defined as < 0.05.

### Flow cytometry

Detection of target cells by FCM, we used PE Mouse Anti-Human CD163(BD Pharmingen™) to sort the macrophages cells. APC Mouse Anti-Human CD326 staining (BD Pharmingen™) to sort the epithelial cells. Staining was carried out according to the manufacturer’s instructions. FCM was performed on a BD FACS Canto II machine. Data were analyzed using FlowJo™ v.10 software.

### RT-PCR

RNA was extracted from RNA extraction from cells after flow cytometry sorting using RNA-easyIsolation Reagent (Vazyme #R701). iScript™ Advanced cDNA Synthesis Kit (Bio-Rad) was performed for reverse transcription, and iTaq™ Universal SYBR^®^ Green Supermix (Bio-Rad) was utilized for the qRT-PCR in accordance with the instructions. The program of qRT-PCR is shown below: initial denaturation for 30 s at 95°C, 40 cycles of denaturation for 5 s at 95°C, and 40 cycles of amplification for 30 s at 60°C. The mRNA expression in cells was represented as the2^^-(ΔΔCt)^, and β-actin was used as the internal reference. The primer sequences are shown in **Table S2**.

## Results

### Diverse cell types in LUAD delineated by single-cell RNA-seq analysis

The study overview is shown in **Figure 1A**. To explore the cellular diversity and transcriptional characteristics in LUAD patients who received anti-PD-1 therapy, we integrated scRNA-seq and bulk-RNA sequencing data associated with anti-PD-1 therapy in LUAD patients from our data and the GEO database (GSE146100) (19). A total of 9 patients were included; of these, 6 patients were treatment-native primary LUAD patients who were PD-L1 positive form our experiment, 1 responder, and 2 non-responders after anti-PD-1 treatment from GEO database (GSE146100). Detailed clinical and pathological information about these patients is provided in (**Supplementary Table 1**). To perform scRNA-seq, cells were dissociated, sorted for viability, and profiled using 10× Chromium Genomics protocols. (**Figure 1A**; STAR Methods). Cells grouped primarily by dataset were mixed after integration by the “Harmony” package, which showed the well-integrated scRNA-seq data (**Figure 1B**). After quality control, a total of 28,376 cells that met the inclusion criteria were selected for the following analysis (including 16,764 from untreated patients 7,896 cells form nonresponders and 3,716 from responders respectively) (**Figure 1B**). Similar to previous studies, tumor cells displayed higher heterogeneity than stromal and immune cells, which grouped together by cell type (20, 21)(**Figure 1C**). By characterizing the canonical cell markers, thirteen major cell types were detected, classified as epithelial cells, immune cell types (CD8 T cells, CD4 T cells, *S100A8+* macrophages, *SPP1+* macrophages, NK cells, B cells, monocytes cells, mast cells), and stromal cell types (fibroblasts and endothelial cells and cell cycling) (**Figure 1D** and **Supplementary Table 2**). In summary, the combination of scRNA-seq data reflected the distribution of the cell type and transcriptional features in the anti-PD-1 treatment cohort of LUAD.

### Cell type-specific aberrant gene expression of responders and nonresponders to anti-PD-1 treatment

**Figure 2:**
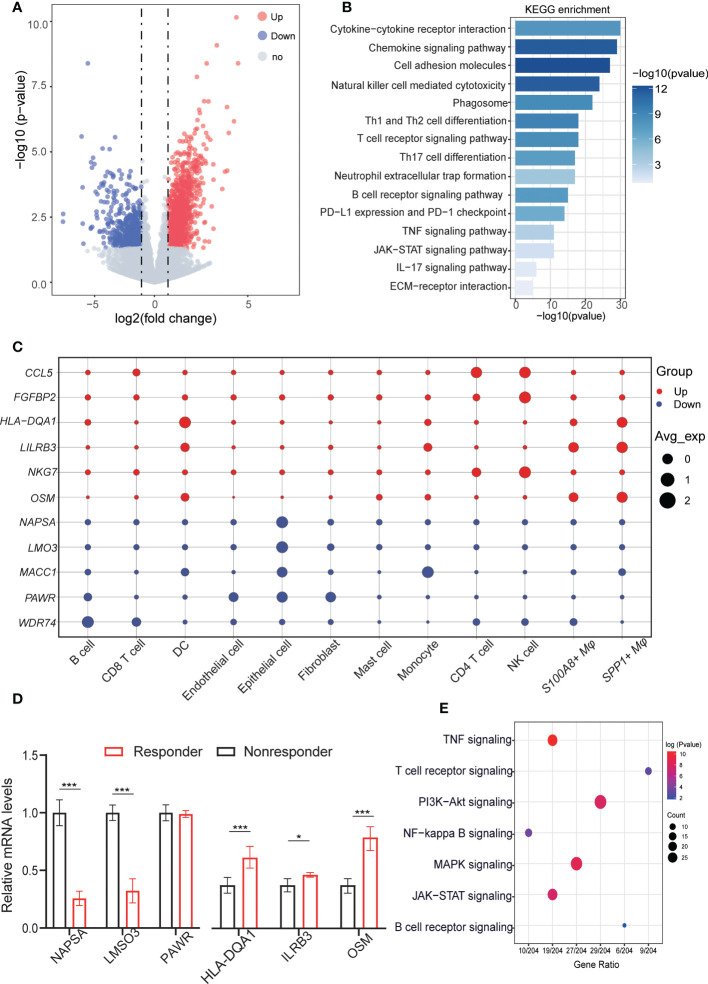
Aberrant gene expression profiles in cell type-specific manners in LUAD with anti-PD-L1 treatment. **(A)** Volcano plot shows the differentially expressed genes (DEGs) between responders and nonresponders analyzed by bulk RNA-seq from datasets. Genes upregulated or downregulated by more than 2-fold are shown in red and blue, respectively. 1488 upregulated genes and 1575 downregulated genes. **(B)** Bar plots displaying the KEGG enrichment of up- or downregulated genes in LUAD patients (responders *vs.* nonresponders). Fisher’s exact tests (two-sided) were performed. A p value < 0.05 was defined as statistically significant. **(C)** Dot plots depicting how the relative expression of particular genes varies at all cell type levels (responders vs. nonresponders). The size displays the values for expression in the cell types, and colors are marked as up- or downregulation in the bulk cohort (red, increased; blue, decreased). **(D)** QRT-PCR validation the representative genes were highly expressed in celltype for responders and nonresponders. **(E)** Dot plot showing the results of KEGG pathways enrichment analyses for the DEGs of responder *vs.* nonresponder at the cellular level. QRT-PCR validation the representative genes were highly expressed in celltype for responders and nonresponders. *p < 0.05; ***p < 0.001.

To simultaneously define gene expression changes at the global and cellular levels, we also performed bulk RNA-seq of responder (CR, complete response; PR, partial response) and non-responder (SD, stable disease; PD, progressive disease) samples after the anti-PD-1 treatment from GEO data (GSE126045) (22). Considering the cut-off criteria (adjusted P-value < 0.05 and |log 2FC| > 1.0), Genes upregulated or downregulated by more than 2-fold are shown in red and blue, respectively. Compared to the nonresponders 1,155 genes were upregulation and 714 genes were downregulated in the responders (**Figure 2A**). To further investigate the molecular function implication of these DEGs (Differentially Expressed Genes), we further adapted the KEGG enrichment analysis, these DEGs were shown to be significantly associated with signaling pathways related to immune and inflammation response pathways related to cancer, such as cytokine-cytokine receptor interaction pathways, chemokine signaling pathway, cell adhesion molecules pathway, natural killer cell mediated cytotoxicity pathways, and PD-L1 expression and PD-1 checkpoint pathway (**Figure 2B**). Our results confirm the previous conclusion that the response to PD-L1 signaling pathways is activated and that NK cells are activated in the responders. In addition, we also found that cell-cell interaction pathways were active in the responder, and we inferred that altered receptor-ligand interactions may play an important role in the microenvironment, leading to different respond to the immunotherapy.

To explore specific aberrancies in the expression of molecules in each cell type in LUAD, we evaluated changes in the expression of representative reported genes at the cell level. To distinguish malignant epithelial cells from nonmalignant cells, we referred to a previous method of tumor cell identification (23). Each epithelial cell was given a malignant or nonmalignant score, the clustering results showed the ability to clearly distinguish of malignant from non-malignant cells (**Figures S1A, B**). Meanwhile, the “inferCNV” results confirmed the difference in copy number variation between malignant cells and non- malignant (**Figure S1C**). As we known, the *NKG7* gene was critical for controlling cancer initiation, growth, and metastasis upregulated in the responder, Similarly, upregulated in NK cells and CD4 T cells at the single cell resolution. C-C motif chemokine 5 (CCL5*)*, Fibroblast growth factor-binding protein 2 (*FGFBP2*), HLA-DQA1 protein (HLA-DQA1), Leukocyte immunoglobulin-like receptor subfamily B member 3 (LILRB3), and Notch ligand osm-11 (OSM) shown to be upregulated in bulk RNA-seq, were upregulated mainly in CD4 T cells, NK cells, SPP1+ macrophage, *S100A8*+ macrophage, whereas its expression was unchanged in other cells. Conversely, Napsin-A (NAPSA*)*, LIM domain only protein 3 (LMO3), Metastasis-associated in colon cancer protein 1 (MACC1*)*, PRKC apoptosis WT1 regulator protein (PAWR), and WD repeat-containing protein 74 (WDR74) gene expression was downregulated in most cell types, but upregulated only in the epithelial cell, fibroblasts cells, and monocytes cells (**Figure 2C**). Next, we validate these results *in vitro* by using fluorescence-activated cell sorting (FACS) and followed by q-RT-PCR (**Figure S2**), Also these results verified by the experiment are consistent with our previous analysis results (**Figure 2D**). The deconvolution of DEGs for bulk RNA-seq in scRNA-seq level, we found that upregulated genes in non-responders were mainly expressed in malignant cells, but upregulated genes in responders were more likely to be distributed in different immune cell types (**Figure S1D**). Then, we further investigated the different pathways in malignant cells within the three groups and the GSVA results showed that significant difference of responder group and nonresponses group existed in IL6/JAK2/STAT3 signaling pathways and inflammatory-response pathways. These differences reflect cellular heterogeneity in gene expression changes, further suggesting that investigating gene expression changes in each cell type in the anti-PD-1 treatment is important (**Figure S1E**).

Next, to identify gene dysregulation in the responders or nonresponders at the level of cell type specificity, the “FindMarkers” algorithm was used to calculate the DEGs between untreated patients, responders, and non-responders for each cell type, top3 genes are marked among all cell types. such as *MMP7* related to ECM components, highly expressed in tumor cells in the responder. Immunomodulatory factors, such as *LY6E*, *IFI27* were both highly expressed in the no responder. *GZMB* plays an important role in T cell- and NK cell-mediated tumor killing and can predict tumor immunotherapy response, which high expression in NK and T cells of the responding population. *FOX* and *GSN* genes were highly expressed in the nonresponses of fibroblasts and endothelial cells, respectively, might serve as a potential target for relieving tumor immunosuppression (**Figure S1F**). In addition, the pathway showed that the differential genes at the cellular level were mainly enriched in immunoregulatory pathways, such as TNF signaling pathway, NF-kappa B signaling pathway, JAK/STAT signaling pathway, in addition to some T cell receptor signaling pathway, B cell receptor signaling pathway plays a role in the activation of T cells or B cells (**Figure 2E**). These results suggest that the immune response of tumor is influenced by multiple cell-cell interactions in the microenvironment and that intercellular receptor-ligand signaling plays an important role. For the DEGs at the tissue and cellular level, we performed differential expression among the untreated patients, responder, and non-responder for each cell type in LUAD, providing a comprehensive molecular expression change of TME in the anti-PD-1 treatment.

### Comparison of cell-cell interactions between untreated patients, responders, and non-responders

**Figure 3:**
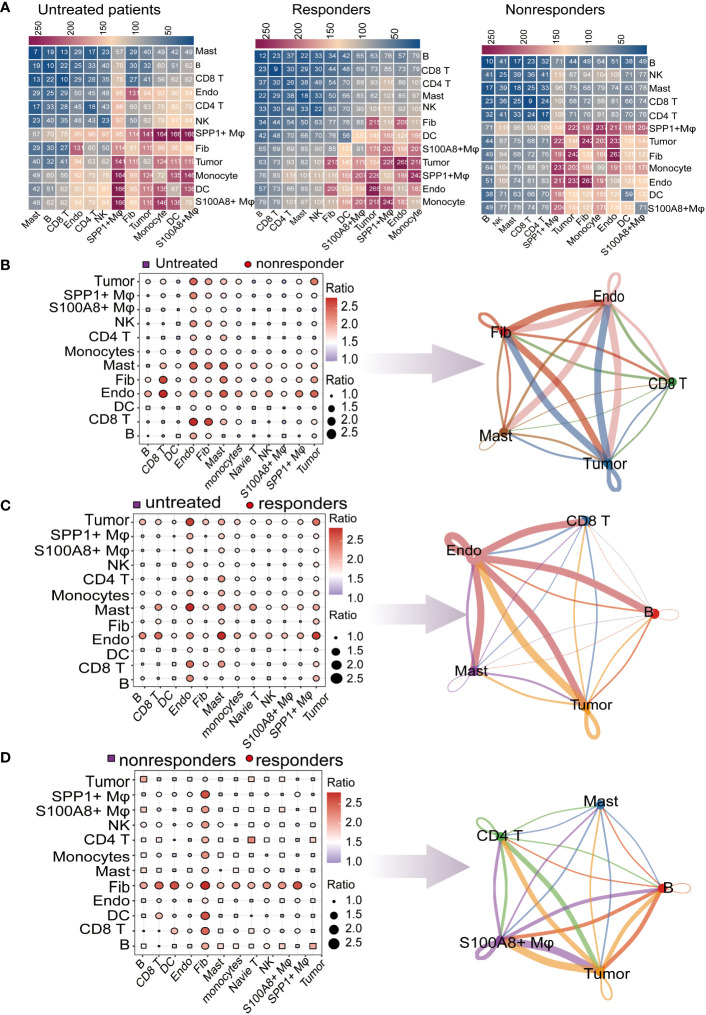
Global analysis of ligand–receptor interaction pairs. **(A)** Detailed network of cell−cell interactions among thirteen cell subsets. Comparison of pretreatment responders and nonresponders. Dot plots depict the changed numbers of putative ligand–receptors in untreated patients compared with nonresponder samples (red, increased; blue, decreased). Cell−cell interactions across all cell pairs in pretreatment responders and pretreatment nonresponders. The top 5 cells with the largest fold change are displayed **(B)** Comparison of cell−cell interactions between pretreatment responders and untreated patients (red, increased; blue, decreased). The top 5 cells with the largest fold change are displayed **(C)** Comparison of cell−cell interactions between responders and untreated patients (red, increased; blue, decreased). The top 5 cells with the largest fold change are displayed. **(D)** Comparison of cell−cell interactions between responders and nonresponders (red, increased; blue, decreased). The top 5 cells with the largest fold change are displayed.

The single-cell analysis results had identified thirteen cell types in the LUAD, to further investigate the potential interactions between different cell types in the TME, the “CellphoneDB” was be used to calculate cell-to-cell interactions (24). there are many interactions with other cell subsets in LUAD, indicating complex and diverse functions in the tumor microenvironment. To comprehend the cell-cell interactions of untreated patients vs. treated (responders or non-responders) in the TME, we calculated the ratio of total interaction frequency within each cell pair between groups (**Figure 3A**). Frequency of intercellular interactions were shown in the **Supplementary Table 3**. In general, the ratios of total scores between cell interactions show endothelial cells, Fibroblasts, Mast cells, Tumor cells, and CD8 T cells were most relatively upregulated in non-responders compared with untreated patients, We inferred that these intercellular interactions changes may be associated with immune unresponsiveness (**Figure 3B**). Compared with untreated patients, we found that CD8 T cells, Endothelial cells, Mast cells, Tumor cells, and B cells had a strong interaction with the responder, which implied that changes in these intercellular interactions may be associated with immune responses (**Figure 3C**). The ratios of total cell-cell interactions between no-responders and responders are shown in (**Figure 3D**). The abundance of cell-cell interactions in Mast cells, CD4 T cells, S100A8+ macrophage, and tumor cells compared to other cells was higher in responders than in non-responders, which indicated that there was more communication between these cell types in the responders.

By calculating the change of interaction ratio between cells in different immunotherapy response patients, the top 5 cell types with the change in interaction ratio were detected. In the subsequent analysis, we will focus more deeply on the molecular mechanisms underlying these changes in cellular interactions in the microenvironment of responders and nonresponders.

### Single-cell transcriptional analysis reveals the cell-cell crosstalk network in responders and nonresponders

To further identify the key mediators of the important cell types cell-cell interactions (CCI) in the anti-PD-1 treatment of LUAD patients, we evaluated the putative crosstalk with the R package “NicheNet” R package based on the expression and downstream targets of ligand-receptor pairs, the “nichenet_seuratobj_aggregate” algorithm can be used for downstream differential cell communication and receptor-ligand regulatory network analysis (25, 26).

**Figure 4:**
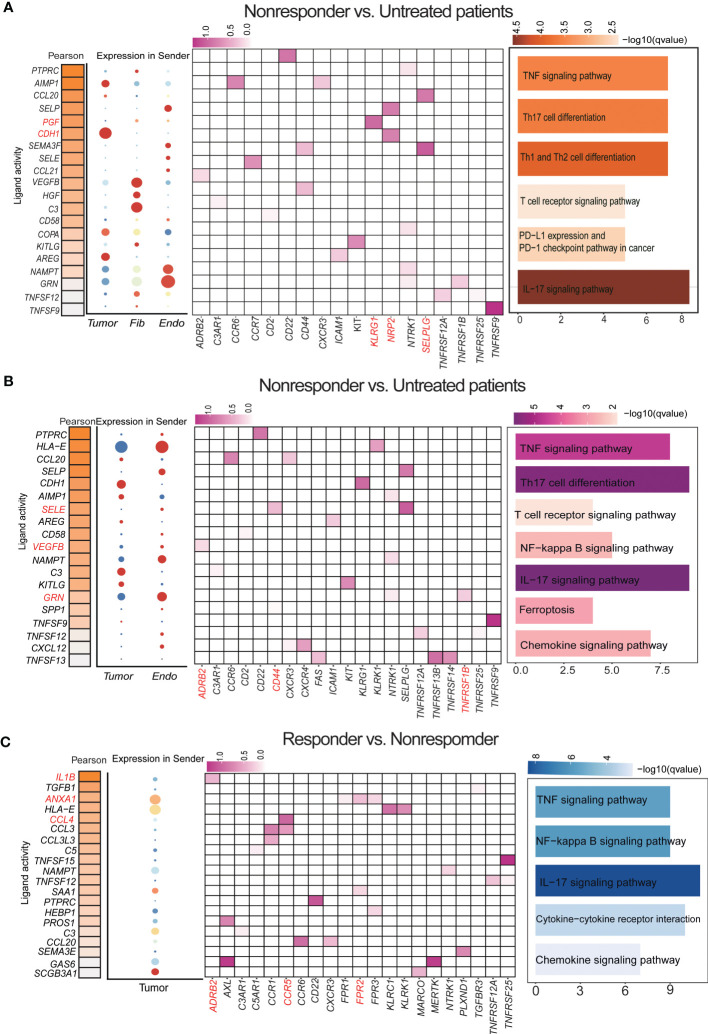
Single-cell transcriptional analysis reveals the cell−cell crosstalk network in LUAD. **(A)** By comparing differences between nonresponders *vs* untreated patient cell interaction receptors and ligands in several key cell types. (a) Ligand activity prediction by “NicheNet” showing the top 20 ligands best predicting all DEGs between sender and receiver cells. (b) The bubble plot shows the expression patterns of the predicted ligands on tumor cells, endothelial cells, and fibroblasts. (c) Ligand–target matrix displayed in LUAD. (d) Representative KEGG pathway enrichment of the predicted target genes expressed. Statistical analysis was performed by Fisher’s test. **(B)** By comparing differences between responders *vs* untreated patients, cell interaction receptors and ligands in several key cell types. **(C)** Cell interaction receptors and ligands in several key cell types were compared between responders and nonresponders.

We designated epithelial cells or stromal cells as the ‘sender’ and immune cells as the “receiver” to elucidate cell–cell regulatory networks. To obtain a comprehensive receptor-ligand molecular profile for immunotherapy response, we first gather statistics on differences in CCI between untreated patients *vs.* no-responder. The result showed the top 20 ligands probable regulating sender cells. There are many significant ligand-receptor pairs between sender and receiver, such as *CDH1-KLRG1, PGF-NRP2, SEMA3F-SELPLG*, and so on. *KLRG1* is an immune checkpoint receptor and a combination blockade of *KLRG1*, and *PD-1* promotes immune control of local and disseminated cancers. Pathway analysis for downstream target genes regulated by receptor ligands shows a high probability that the target genes belonged to the TNF signaling pathway, Th17 cell differentiation, T cell receptor signaling pathways, extracellular matrix pathways, PD-L1 expression and PD-1 checkpoint pathway in cancer, and the chemokine signaling pathway (**Figure 4A**). The results of differential cellular communication implicate the role of these receptor-ligand pairs in immune resistance after immunotherapy. Second, we compare CCI untreated patients *vs.* no-responder. The result showed that *SELE-CD44*, *VEGFB-ADRB2*, *GRN-TNFRSF1B*, and so on. Pathway analysis of downstream target genes showed Th17 cell differentiation, chemokine signaling pathway, and NF−kappa B signaling pathway were enriched (**Figure 4B**). Finally, the same method is used for statistical CCI responder *vs.* no-responder. The result showed that *IL1B-ADRB2*, *CCL4-CCR5*, *FPR2-ANXA1*, and so on. Pathway analysis indicated IL−17 signaling pathway, NF−kappa B signaling pathway, and TNF signaling pathway were enriched in the downstream target genes (**Figure 4C**).

All of us provide a comprehensive characterization of LR-Pairs of responder or no-responder after anti-PD-1 therapy in LUAD, Ligand-receptor interactions (LRIs) between different cell types in the TME play a vital role in the occurrence and development of anti-PD-1 treatment responses.

### Molecular characteristics of different molecular subgroups of LUAD and their association with clinical outcomes based on LR-pairs

**Figure 5:**
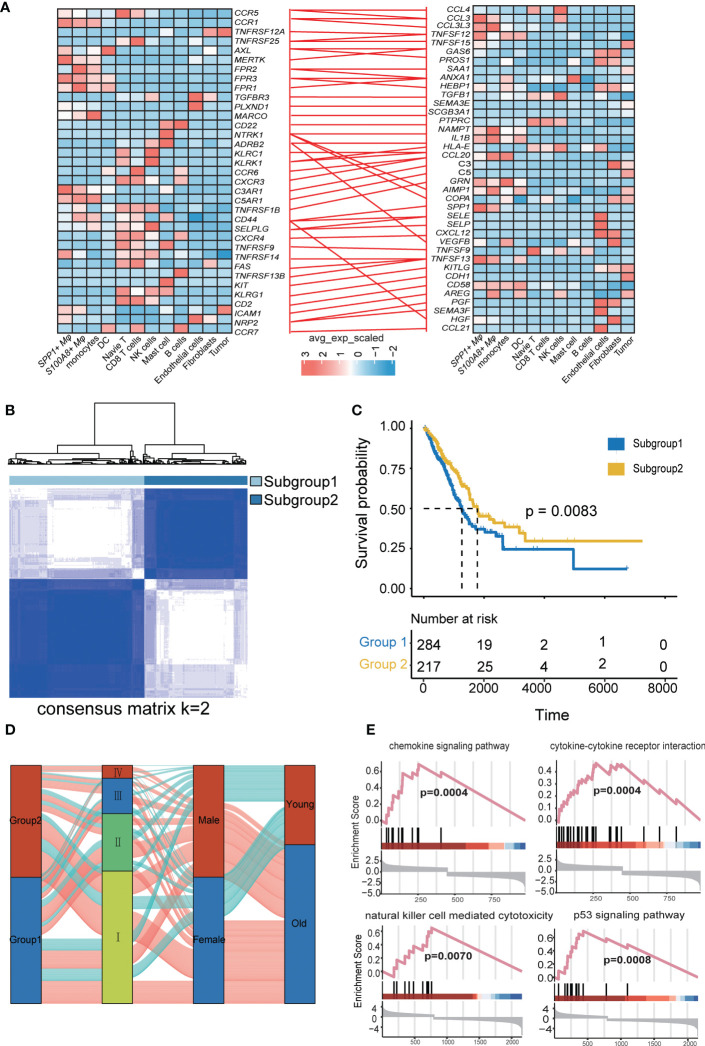
Identification and validation of the LRI-based classification of LUAD patients. **(A)** The ligand−receptor interaction pairs. The color of the line between the ligand receptors represents differential LRIs between untreated patients and different treatment responses. **(B)** Consensus clustering matrix for the ideal cluster size in the TCGA LUAD cohort, *k* = 2, which was represented by the matrix. **(C)** Kaplan−Meier survival analyses of the patients in subgroups 1 and 2 of LUAD in the TCGA showed that subgroup 1 patients had a worse OS than subgroup 2 in the LUAD patients. **(D)** Alluvial diagram depicting the relationships between LRIs and clinicopathological characteristics in two subgroups of LUAD patients in the TCGA cohorts. **(E)** GSEA of the chemokine signaling pathway, cytokine−cytokine receptor interaction pathway, natural killer cell-mediated cytotoxicity pathway, and p53 signaling pathway between the two subgroups.

To further understand the ligand-receptor (LR) pairs related to immunotherapy, we combined LR pairs related to immunotherapy response or immunotherapy resistance (**Figure 5A**). All of us provide a comprehensive characterization of LR-Pairs of responders or nonresponders after anti-PD-1 therapy in LUAD. Therefore, by combining three LR-Pairs datasets we analyzed the expression profiles of 73 LR-related genes in 501 LUAD samples from the TCGA database to construct consensus clustering. Based on their cumulative distribution function and function delta area, we chose *k* = 2, where LR-related genes appeared to be stably clustered, and then we obtained two subgroups designated subtype 1 and subtype 2 (**Figure 5B**). Two molecular subgroups showed significant differences in prognosis (p < 0.001), and subtype 2 was associated with a better prognosis (**Figure 5C**). Next, we compared the DEGs between subtype 1 and subtype 2 and found that the immune response and inflammatory pathways such as the chemokine signaling pathway, cytokine-cytokine receptor interaction pathways, Natural killer cell mediated cytotoxicity pathways, and p53 signaling pathway were more active in subtype 2, implying that subtype 2 might be immune infiltration tumor and subtype 1 is likely to be an immunosuppressive tumor. Taken together, tumor immunotyping can be used to predict the prognosis of LUAD patients and may provide therapeutic strategies for improving the clinical benefit of cancer immunotherapy (**Figures 5D, E**).

### The immune landscape of LUAD patients in different tumor subgroups

**Figure 6:**
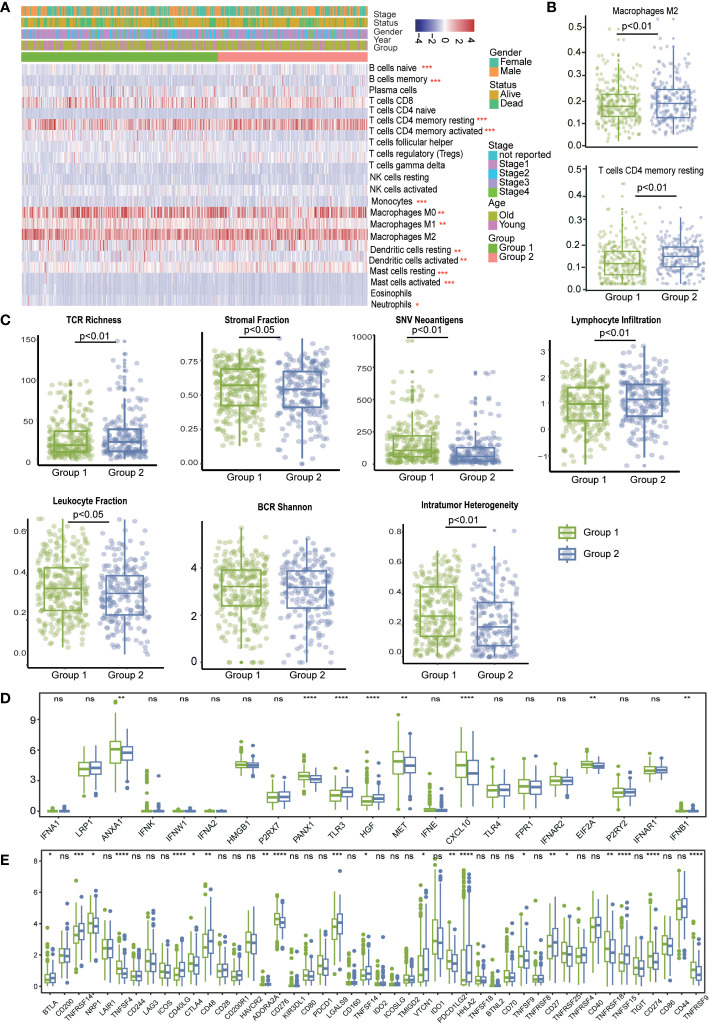
The immune landscape in distinct LRI-related molecular patterns in LUAD. **(A)** “CIBERSORT” analysis identifying the relative infiltration level of immune cell populations in two LRI subgroups of LUAD samples in the TCGA cohort. **(B)** Differences in regulatory T-cell and MDSC proportions among distinct LRI subgroups in TCGA cohorts. **(C)** The relative distributions of the leukocyte fraction, stromal fraction, SNV neoantigens, indel neoantigens, TCR Shannon, BCR Shannon, and ITH scores were compared among the two LRI clusters. **(D, E)** Association between immune subgroupsand ICPs and ICD modulators. a, b; Differential expression of ICP genes among the two LUAD subgroups. ns p ≥0.05; *p < 0.05; **p < 0.01; ***p < 0.001; ****p < 0.0001.

To investigate the relationship between LRIs and immune infiltration in LUAD. Thus, we used the “CIBERSORT” method for cell-type deconvolution (27), the Wilcoxon- test to compare the distribution of 28 infiltrating immune in different LUAD molecular subgroups (**Figure 6A**). We found that macrophages M2 and T cells CD4 memory resting was markedly elevated in subtype 2, which was concordant with previous observations linking subtype 1 to an immunosuppressive phenotype (**Figure 6B**). In addition, we explored the immune characteristics in distinct LR-Pairs subgroups. As shown in (**Figure 6C**), both TCR richness leukocyte and stromal fractions were increased in Cluster 2. Cluster 1 had the highest SNV (single-nucleotide variant) neoantigens, Indel neoantigens, and ITH (Intra-tumor heterogeneity), while Cluster 2 was defined by the lowest neoantigens and ITH. Immunomodulators play a critical role in shaping TME and cancer immunotherapy. Therefore, to further investigate the complex crosstalk of immunomodulators, immune infiltration, and LR-Paris genes, we explored the expression of immunomodulators in the different subgroups. Immune checkpoint molecules, like *CTLA-4*, *TIM-3*, and *PD-1* are negative regulators of immune responses to avoid immune injury. Checkpoint regulators are thought to actively participate in the immune defense of infections, prevention of autoimmunity, transplantation, and tumor immune evasion. Given the importance of immune checkpoints (ICPs) and immunogenic cell death (ICD) modulators in cancer immunity, we next analyzed their expression levels in the different subgroups, *ANXA1*, *PANXA1*, *MET*, *CXCL10*, *ELF2A* were overexpressed in the subgroup1, While *TLR3*, *HGF* overexpressed in subgroup2. For instance, we identified a number of molecules associated with immune checkpoint inhibitors like *CTLA4*, *ADORA2A*, *TNFSF9*, *TNFRSF18*, *CD274*, *TNFRSF9* were significantly upregulated in subgroup1 in the TCGA cohort. This result confirmed that subgroup1 might be an immunosuppressive tumor subtype (**Figures 6D, E**).

Taken together, our comprehensive analysis revealed that the LR-Pairs subgroups were significantly correlated with the patient prognosis and TME characteristics, which might provide new insights into LUAD anti-PD-1 therapy.

### Establishment and validation of the LRI-based prognostic risk score model for overall survival in LUAD

**Figure 7:**
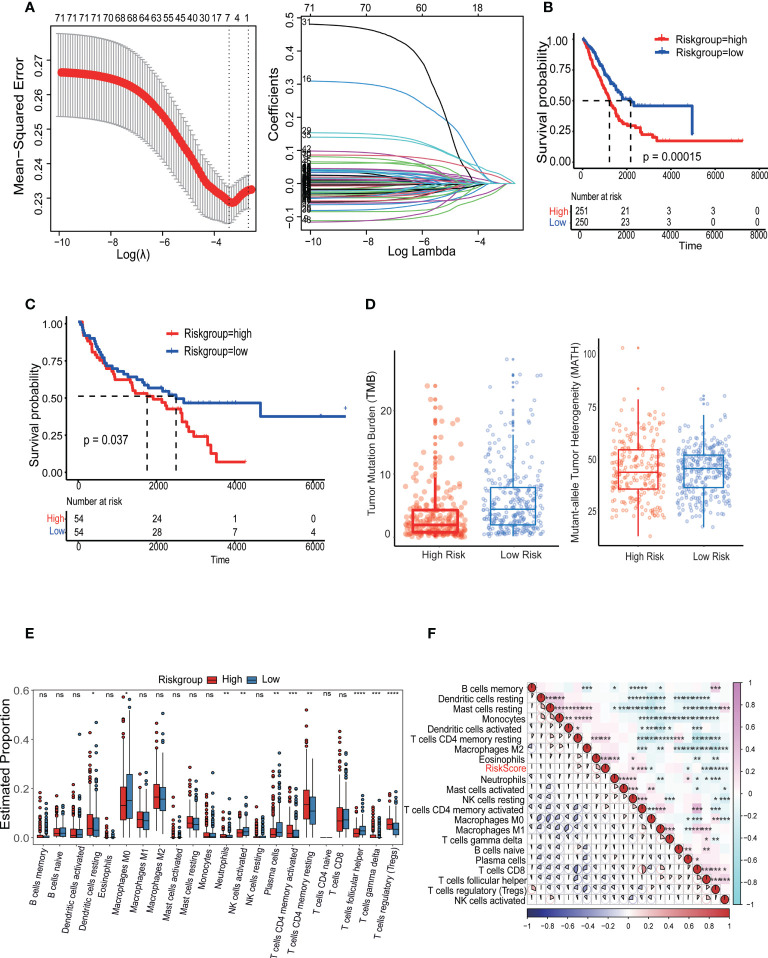
Identification and validation of the LRI-based classification of LUAD patients. **(A)** Construction of the cellular senescence score (CSS) and the impact of CSS on the clinical outcome of LUAD patients. A 336 cellular senescence cluster-related differentially expressed genes (DEGs) among the three cellular senescence clusters are shown in the Venn diagram. **(B, C)** Kaplan–Meier curves of L-R scores for patients in the high and low groups in the TCGA-LUAD cohort. **(D)** The tumor mutation burden (TMB) score and mutant-allele tumor heterogeneity (MATH) in the different subgroups of LUAD. **(E)** Analysis of the immune cell scores between the different L-R score groups using the CIBERSORT algorithm. **(F)** Correlation between the L-R score and immune cell score. ns p≥0.05; *p< 0.05; **p<0.01; ***p<0.001; ****p<0.0001.

The LASSO Cox algorithm was used to identify the most robust prognostic genes among the 73 candidate LR-related genes. Overall, by performing least absolute shrinkage and selection operator (LASSO) Cox regression analysis, the 7 genes (*TNFSF13*, *AXL*, *KLRK1*, *FAS*, *PROS1*, and *CDH1*) that met the criteria of P < 0.05 were retained for further analysis, Furthermore, the LR-pairs scoring model was constructed using the 7 LR-pairs (**Figure 7A**). Based on two independent cohort KM survival analyses, it was verified that patients in the high-risk group had significantly worse OS than those in the low-risk group (p=0.00015, p=0.037) (**Figures 7B, C**). To investigate the molecular gene-mutation characteristics between the LR-score high and LR-score low risk groups, we first compared the mutation frequency between the two groups. We used mutect2 to calculate the mutant-allele tumor heterogeneity score (MATH) and tumor mutation burden (TMB) score to assess tumor heterogeneity and somatic mutation rates. As shown in, the low-risk group showed significantly higher TMB than the high-risk group, we hypothesized that samples within the low-risk group may have a better benefit from immunotherapy (**Figure 7D**). The mutation frequencies of the *TTN*, *CSMD3* and *ZFHX4* genes varied significantly among subgroups, with higher mutation frequencies observed in the high-risk group (**Figures S3A, B**). Furthermore, significant disparities in immune cell infiltration levels were observed among the L-R score groups, Additionally, there was significant infiltration of T cells regulatory (Tregs), with the high LR score group displaying much higher infiltration levels than the low LR score group (**Figures S3C**, **7E**). The association between the 22 immune cell scores and the L-R risk score was examined using Pearson’s correlation coefficient. The findings show that the L-R score was significantly positively connected with T cells CD4 memory resting, Eosinophils, and T cells CD4 memory activated but negatively correlated with regulatory T cells and T cells follicular helper (**Figure 7F**). In summary, these findings suggest that patients with low risk with longer survival time, high TMB score and MATH score, hinted at a possible benefit in the immunotherapy.

### The relationship between LR-score and immunotherapy

**Figure 8:**
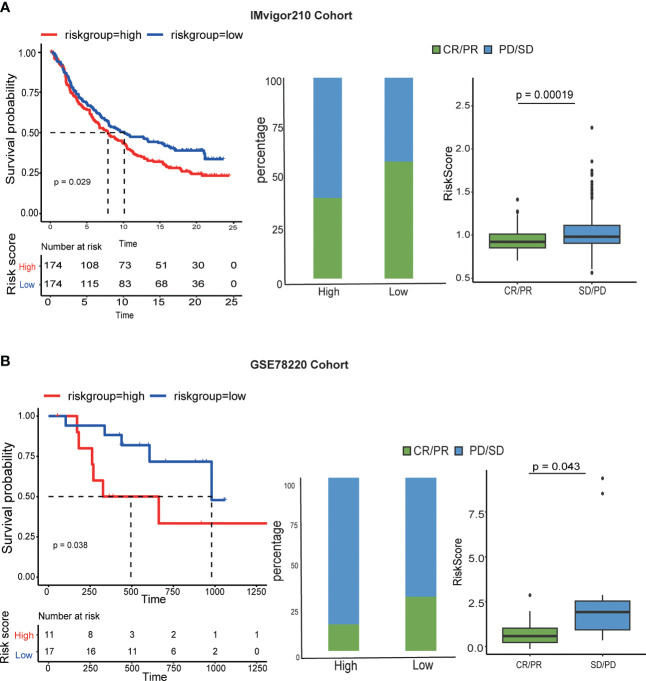
Correlation between the LRI score and response to anti-PD-L1 immunotherapy. **(A)** (a) Differences in L-R scores between responders and nonresponders in the IMvigor210 cohort. (b) The proportion of patients responding to immunotherapy in the high and low L-R score groups in the cohort. (c) Prognostic differences between the high and low L-R score groups in the cohort. **(B)** (a) Prognostic differences between the high and low L-R score groups in early-stage patients in the GES78220 cohort. (b) Prognostic differences between the high and low L-R score groups in advanced patients in the cohort. (c) L-R score differences between responders and nonresponders in the cohort.

To ascertain the relationship between the LRIs-score and immunotherapy, it was examined how well the LR-score could forecast a patient’s reaction to immune checkpoint blockade (ICB) immunotherapy efficacy. In the anti-PD-L1 IMvigor210 cohort, 348 patients showed various levels of response to anti-PD-L1 receptor blockers, including complete remission (CR), partial response (PR), stable disease (SD), and progressing illness (PD) (28). The LR-score was higher in SD/PD patients than CR/PR individuals. A comparison of percentages between patients with high or low LRIs score revealed that high-risk score patients have fewer effective therapies. Further survival studies revealed that, in patients undergoing immunotherapy, the LRIs score was significantly correlated with overall survival(p=0.029) (**Figure 8A**). Additionally, in a different cohort of GES78220 anti-PD-1 patients, those with SD/PD had a higher LR score than those with CR/PR (29). Additionally, percentage data comparing patients with high and low LRIs score revealed that those with low LRIs score had superior treatment results, therapeutic benefits, and longer overall life (p = 0.038) (**Figure 8B**). These findings suggested the reliability of the immunotherapy efficacy evaluation and prognostic model, which can be applied to diverse LUAD patients.

## Discussions

It is well known that the TME is composed of a complex cell−cell interaction regulatory network by cell type, and it plays a crucial role in the progression and response to treatment of tumors, including drug resistance, immune escape, and tumor metastasis (30). Although current immune checkpoint blockade (ICB) therapy aimed at targeting receptor−ligand interactions has been successful, there are still quite a few patients who cannot benefit from it (31, 32). It is essential to understand the mechanism of intracellular communication and find new therapeutic targets. scRNA-seq is an effective method to analyze the TME cell composition and crosstalk between cell types, and it has provided a large number of resources LRIs in previous studies. Here, the bulk-Seq and scRNA-Seq data of LUAD were integrated for further analysis by comparing the different cell−cell interactions in untreated patients, responders, and nonresponders to anti-PD-1 treatment. A few crucial LRIs were detected that revealed the complexity and plasticity of the TME. Next, these LRIs also provide the foundation for two molecular subtyping models, prognostic evaluation, and immunotherapy utility. These findings contribute to a better understanding of cell−cell communication in the TME of immunotherapy in LUAD and offer new therapeutic possibilities.

The mechanisms of tumor immune resistance or response are very complex and involve multiple aspects, such as genes, metabolism, and inflammation (33). Due to cell type heterogeneity, bulk RNA-seq results might not identify whether these changes are intrinsic molecular changes or simply reflect changes in the proportions of cell types. We not only evaluated changes in the expression of representative reported genes in the cell layer but also identified gene dysregulation in responders or nonresponders at the level of cell type specificity. Interestingly, the upregulated genes in the nonresponders were primarily expressed in epithelial cells, but upregulated genes in the responders were more likely to be distributed in different immune cell types. We speculate that epithelial cells play a key role in immune resistance in LUAD and might target epithelial cells that can relieve immunosuppression and provide treatment benefits in the future. In addition, these results confirmed that some genes were dysregulated in most cell types. We further understand the TME, and the interactions between tumors and other cells could provide important insights into tumor biology and help build reliable prognostic and predictive models.

Single-cell RNA technology applied to immunotherapy response has become increasingly prominent in oncology in recent years, with remarkable results also being achieved in cancer (17, 34). Several studies have identified crucial cell types that respond to immunotherapy. Temporal scRNA-seq and T-cell receptor (TCR) sequencing analyses have shown that *Texp* (precursor-exhausted T cells) tend to accumulate in lung cancer and are significantly reduced after PD-L1 therapy (35). In this study, we focused on LRIs mediated by cell−cell communication with different immunotherapy responses. We identified several different cell−cell interaction pairs based on untreated patients, responders, and nonresponders, and we found that CD8 T cells/endothelial cells/mast cells/tumor cells/B cells had a stronger interaction in responders than in untreated patients. The abundance of cell−cell interactions in mast cells/CD4 T cells/S100A8+ macrophages/tumor cells compared to other cells was higher in responders than in nonresponders. Interactions between these cell types play a key role in immunotherapy response and nonresponse to anti-PD-1 therapy. Furthermore, to further identify the key mediators of the important cell types and cell−cell interactions in the anti-PD-1 treatment of LUAD patients, we further investigated LRIs and the regulation of downstream genes. Together, these results provide in-depth insights into the mechanisms underlying response, provide important insights into tumor biology, and help build reliable prognostic and predictive models.

Currently, to further confirm the effectiveness of our typing analysis, data on the L-R pairs subgroups of TCGA-LUAD data, wherein LUAD samples were divided into two molecular subgroups based on 73 LR-related genes. The results showed that the two molecular subgroups showed significant differences in prognosis (p < 0.001), and subtype 2 was associated with a better prognosis than subtype 2. Pathway analysis of DEGs between subgroup 1 and subgroup 2 showed that the immune response and inflammatory pathway were more active in subgroup 1, implying that these important receptor−ligand pairs can delineate the TME well. In addition, this study examined the relationship between immunotherapy and the L-R score to assess the benefit of the L-R score in different immunotherapy cohorts. The results showed that patients with higher L-R scores showed less favorable responses to immunotherapy and poorer survival status. This suggests that immunotherapy could benefit patients with a lower L-R score. Additionally, the significant differences in survival between the high and low LR score groups in both immunotherapy cohorts illustrate its association with immunotherapy.

This new technology offers new opportunities for the analysis of cell−cell interactions. Current posttranslational modification (PTM) studies highlight the importance of glycosylation, lipid modification, and ubiquitination in checkpoint function (36). However, our understanding of checkpoint PTMs is still very limited. Spatial transcriptomics (ST) ligand−receptor analysis can analyze the spatial distribution characteristics of LRIs, reveal the interaction between cells in spatial niches, and finally infer the spatial regulatory network between different cell types (37). The application of these technologies will make our results more comprehensive in the future and offer multidimensional information to understand the molecular mechanism of response and nonresponse after anti-PD-L1 anti-PD-1 treatment.

In summary, multiomics integrative analysis is a valuable and powerful tool that provides a complementary and more comprehensive understanding of LUAD and offers an opportunity to expedite the translation of basic research to more precise diagnosis and treatment in the clinic.

## Data availability statement

The original contributions presented in the study are included in the article/**Supplementary Material**. Further inquiries can be directed to the corresponding author.

## Ethics statement

This study was approved by the Research Ethics Committee of West China Hospital, Sichuan University. Ethical approval for this study was provided by the Ethics Committee on Biomedical research, West China Hospital of Sichuan University on 18/12/2020.

## Author contributions

LZ conceived the project and designed the experiments, SM performed bioinformatic analysis. SM drafted the manuscript. SM, LYZ, YY, and ZL contributed to the experiments and analyzed the data. All authors contributed to the article and approved the submitted version.

## Supplementary material

Supplementary Figure 1 Aberrant gene expression profiles in cell type-specific manners in LUAD with anti-PD-L1 treatment. **(A)** Volcano plot shows the differentially expressed genes (DEGs) of the tumor compared with the adjacent tissue analyzed by bulk RNA-seq datasets. **(B)** Malignant scores (x-axis) and nonmalignant scores are distributed on a scatter plot (y-axis). Color coding is used to represent density and assign each point to a cell. **(C)** Large-scale CNVs for each cell type are displayed on the heatmap. Tumor cells are represented in the bottom heatmap, and the expression levels for nonmalignant cells are plotted in the top heatmap, with genes arranged across the chromosomes from left to right. **(D)** Upregulated and downregulated gene scores were assessed in untreated patients, nonresponders, and responders with differentially expressed genes. **(E)** Pathway activities were scored for epithelial cells in LUAD patients using the “irGESA” algorithm. **(F)** Dotplot showing the relative expression of representative reported immune response genes and other representative genes in each cell type in nonresponder *vs.* untreated patients, responder *vs.* untreated patients, and responder *vs.* nonresponder.

Supplementary Figure 2 Flow cytometry analysis the cell type in LUAD with anti-PD-L1 treatment. **(A)** APC Mouse Anti-Human CD326 staining (BD Pharmingen™) to sort the epithelial cells in the responders and non-responders (Left: APC-Black; Right: APC-CD326) **(B)** PE Mouse Anti-Human CD163(BD Pharmingen™) to sort the macrophage cells in the responders and non-responders (Left: PE-Black; Right: PE-CD163).

Supplementary Figure 3 Correlation between L-R score mutation characteristics and immune-related characteristics. **(A, B)** Heatmap showing the top 20 gene mutation frequencies in the high- and low-risk groups in LUAD. **(C)** Analysis of the immune cell scores between the different L-R score groups using the CIBERSORT algorithm, distribution, and expression of the 22 types of immune cells in the TCGA-LUAD cohort.
