# Supplementary material for: Receptor–ligand pair typing and prognostic risk model of response or resistance to immune checkpoint inhibitors in lung adenocarcinoma
Source: Front Oncol. 2023 Apr 19;13:1170942. doi: 10.3389/fonc.2023.1170942 (PMC10154538; doi:10.3389/fonc.2023.1170942)
Supplement: Supplementary Figure 1 — Aberrant gene expression profiles in cell type-specific manners in LUAD with anti-PD-L1 treatment. (A) Volcano plot shows the differentially expressed genes (DEGs) of the tumor compared with the adjacent tissue analyzed by bulk RNA-seq datasets. (B) Malignant scores (x-axis) and nonmalignant scores are distributed on a scatter plot (y-axis). Color coding is used to represent density and assign each point to a cell. (C) Large-scale CNVs for each cell type are displayed on the heatmap. Tumor cells are represented in the bottom heatmap, and the expression levels for nonmalignant cells are plotted in the top heatmap, with genes arranged across the chromosomes from left to right. (D) Upregulated and downregulated gene scores were assessed in untreated patients, nonresponders, and responders with differentially expressed genes. (E) Pathway activities were scored for epithelial cells in LUAD patients using the “irGESA” algorithm. (F) Dotplot showing the relative expression of representative reported immune response genes and other representative genes in each cell type in nonresponder vs. untreated patients, responder vs. untreated patients, and responder vs. nonresponder. [file DataSheet_1.zip › Supplementary Figure legend.docx]

**Figure S1. Aberrant gene expression profiles in cell type-specific manners in LUAD with anti-PD-L1 treatment
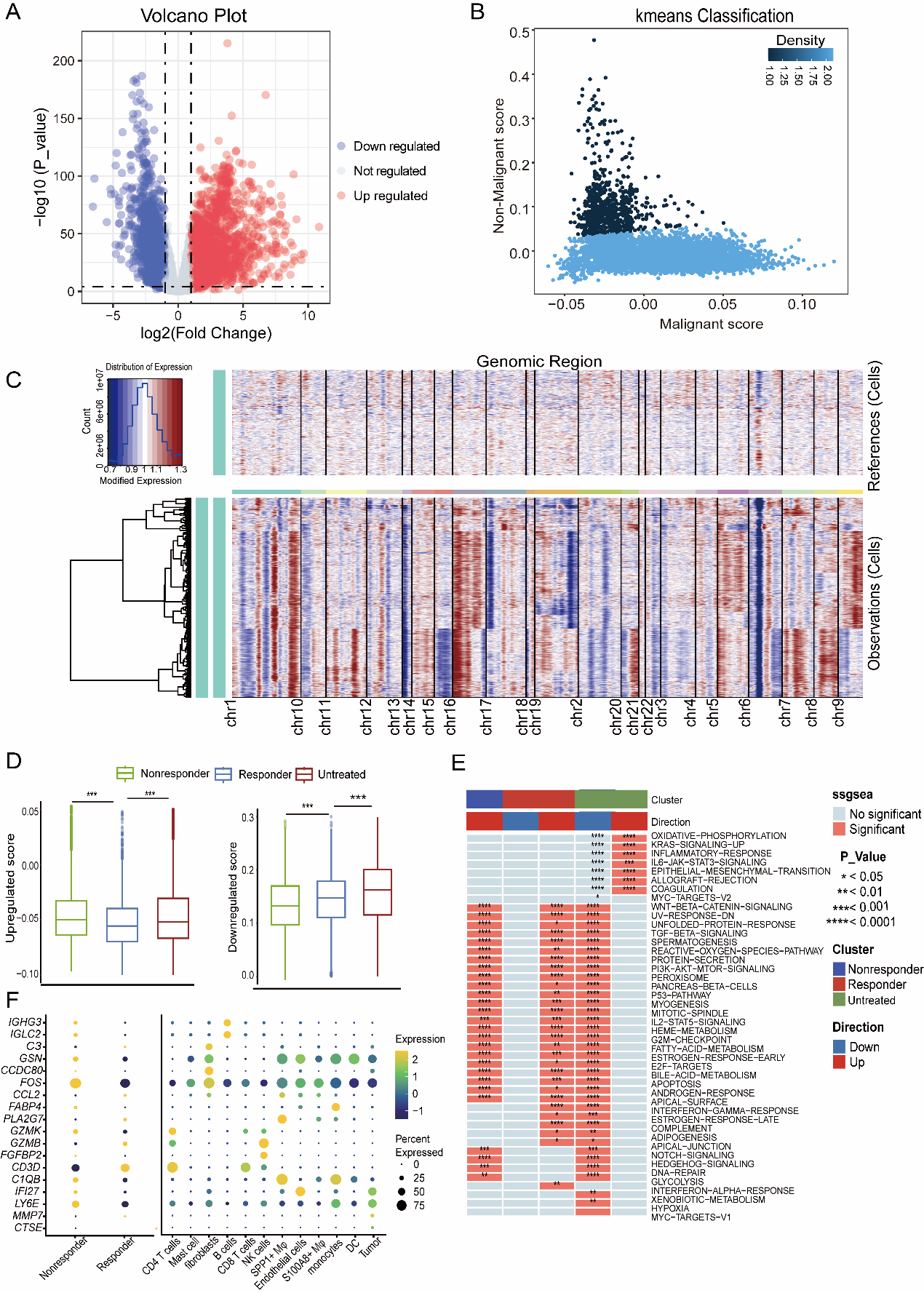
**

**(A)** Volcano plot shows the differentially expressed genes (DEGs) of the tumor compared with the adjacent tissue analyzed by bulk RNA-seq datasets.

**(B)** Malignant scores (x-axis) and nonmalignant scores are distributed on a scatter plot (y-axis). Color coding is used to represent density and assign each point to a cell.

**(C)** Large-scale CNVs for each cell type are displayed on the heatmap. Tumor cells are represented in the bottom heatmap, and the expression levels for nonmalignant cells are plotted in the top heatmap, with genes arranged across the chromosomes from left to right.

**(D)** Upregulated and downregulated gene scores were assessed in untreated patients, nonresponders, and responders with differentially expressed genes.

**(E)** Pathway activities were scored for epithelial cells in LUAD patients using the “irGESA” algorithm.

**(F)** Dotplot showing the relative expression of representative reported immune response genes and other representative genes in each cell type in nonresponder *vs.* untreated patients, responder *vs.* untreated patients, and responder *vs.* nonresponder.

**Figure S2. Flow cytometry analysis the cell type in LUAD with anti-PD-L1 treatment.**
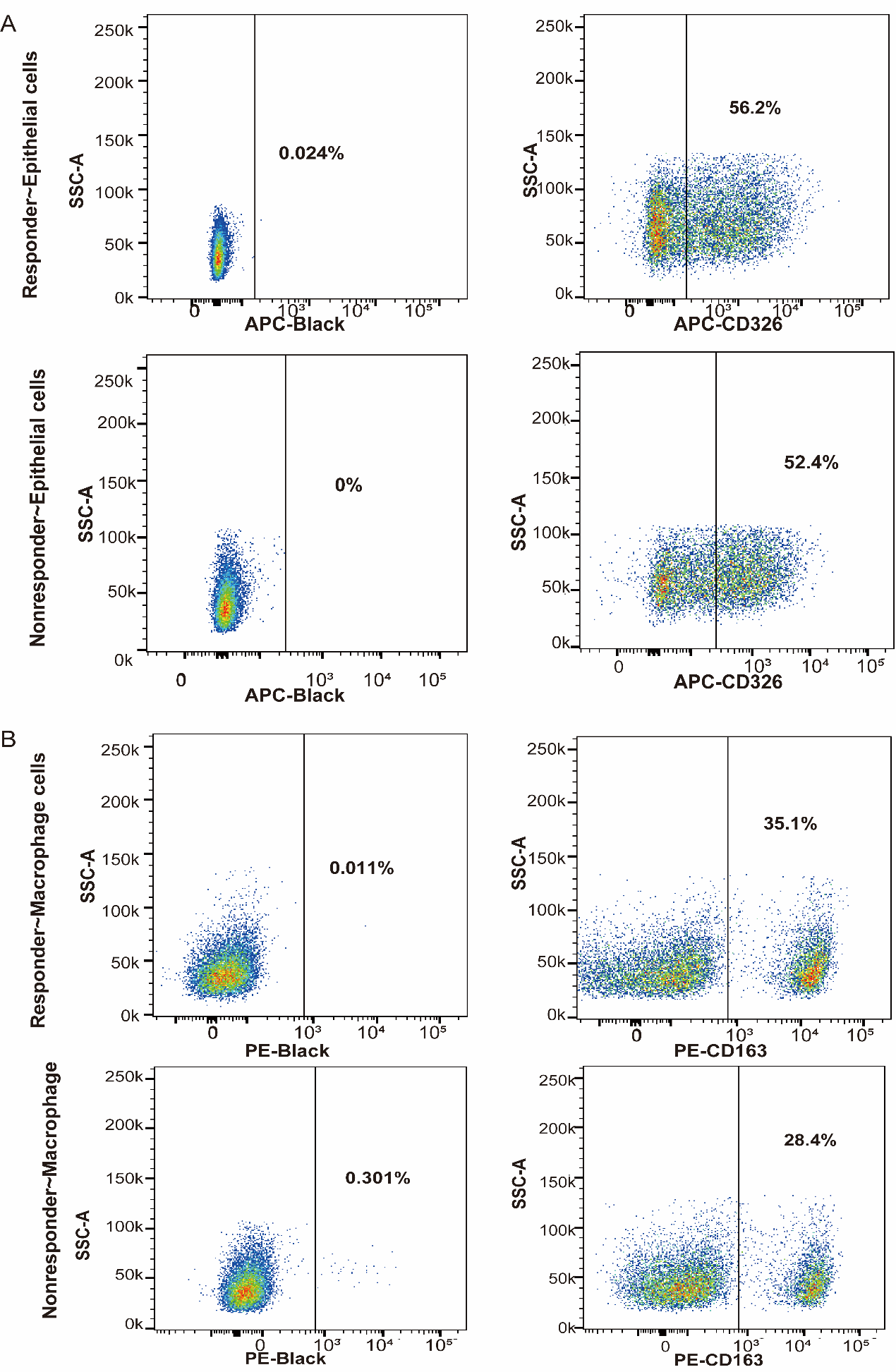


1. APC Mouse Anti-Human CD326 staining (BD Pharmingen™) to sort the epithelial cells in the responders and non-responders (Left: APC-Black; Right: APC-CD326)
2. PE Mouse Anti-Human CD163(BD Pharmingen™) to sort the macrophage cells in the responders and non-responders (Left: PE-Black; Right: PE-CD163).

**Figure S3. Correlation between L-R score mutation characteristics and immune-related characteristics.
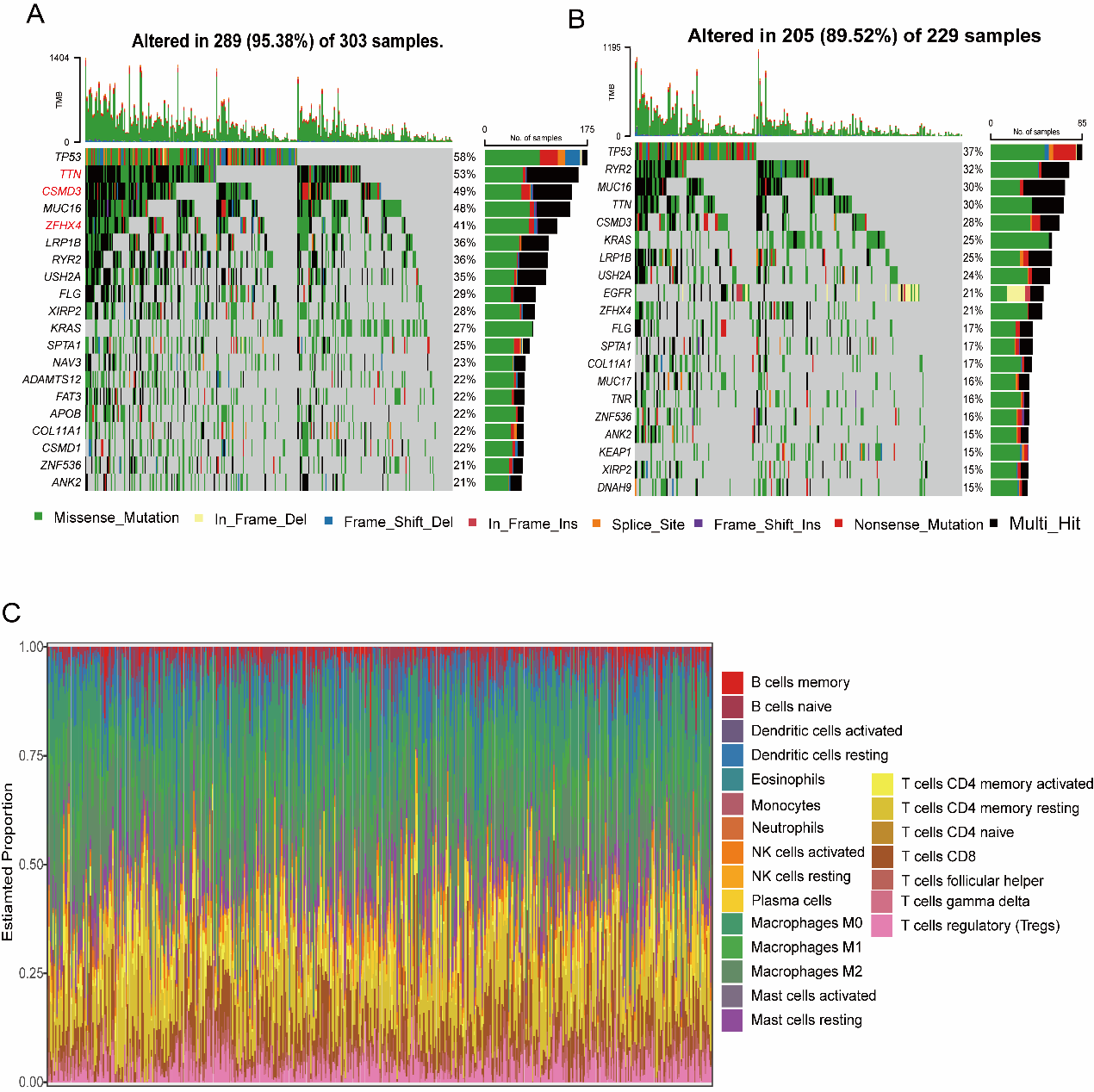
**

**(A)(B)** Heatmap showing the top 20 gene mutation frequencies in the high- and low-risk groups in LUAD.

**(C)** Analysis of the immune cell scores between the different L-R score groups using the CIBERSORT algorithm, distribution, and expression of the 22 types of immune cells in the TCGA-LUAD cohort.
