# Supplementary figures and images for: Receptor–ligand pair typing and prognostic risk model of response or resistance to immune checkpoint inhibitors in lung adenocarcinoma
Source: Front Oncol. 2023 Apr 19;13:1170942. doi: 10.3389/fonc.2023.1170942 (PMC10154538; doi:10.3389/fonc.2023.1170942)

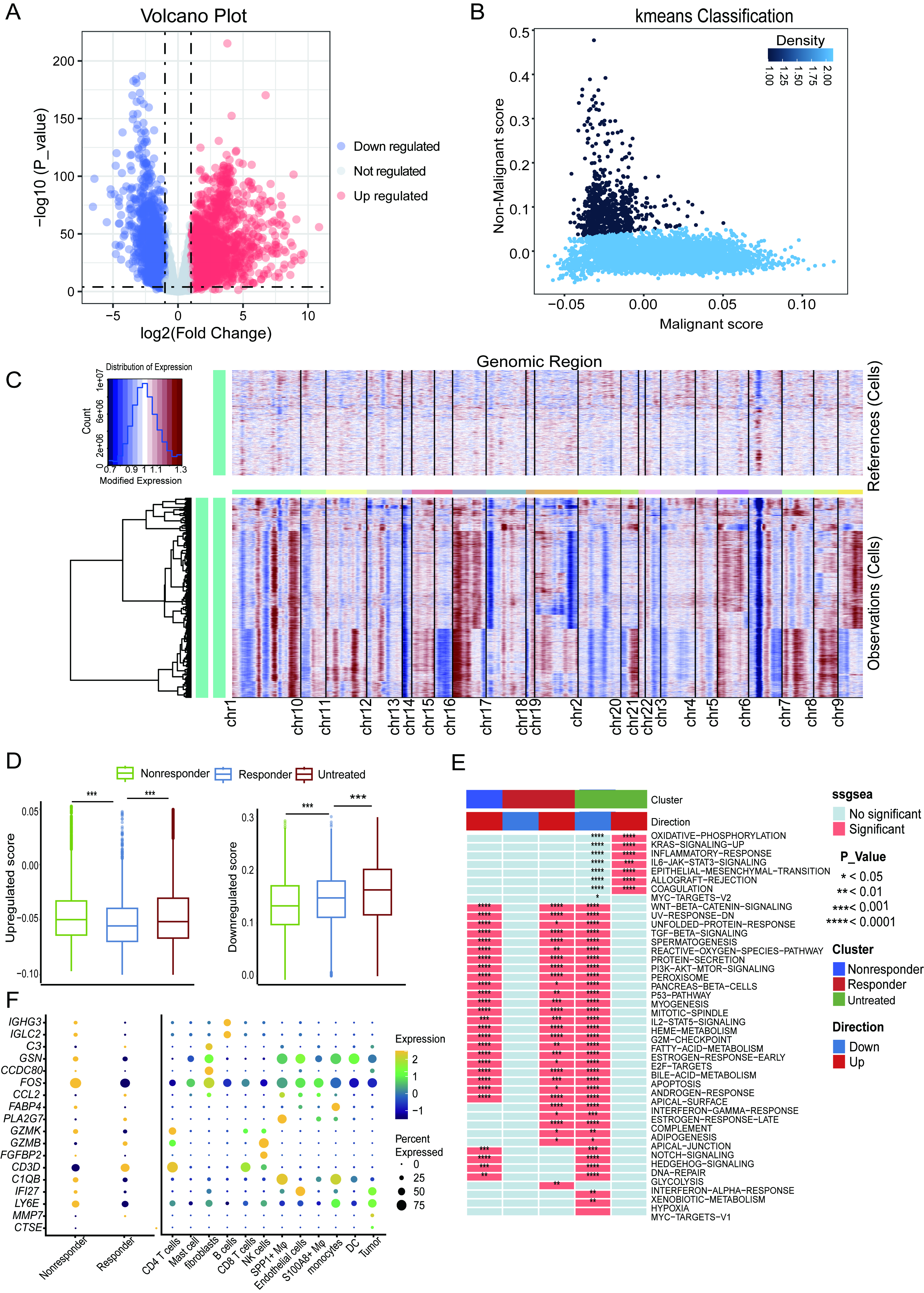

Supplement: Supplementary Figure 1 — Aberrant gene expression profiles in cell type-specific manners in LUAD with anti-PD-L1 treatment. (A) Volcano plot shows the differentially expressed genes (DEGs) of the tumor compared with the adjacent tissue analyzed by bulk RNA-seq datasets. (B) Malignant scores (x-axis) and nonmalignant scores are distributed on a scatter plot (y-axis). Color coding is used to represent density and assign each point to a cell. (C) Large-scale CNVs for each cell type are displayed on the heatmap. Tumor cells are represented in the bottom heatmap, and the expression levels for nonmalignant cells are plotted in the top heatmap, with genes arranged across the chromosomes from left to right. (D) Upregulated and downregulated gene scores were assessed in untreated patients, nonresponders, and responders with differentially expressed genes. (E) Pathway activities were scored for epithelial cells in LUAD patients using the “irGESA” algorithm. (F) Dotplot showing the relative expression of representative reported immune response genes and other representative genes in each cell type in nonresponder vs. untreated patients, responder vs. untreated patients, and responder vs. nonresponder. [file DataSheet_1.zip › FigureS1.tif]

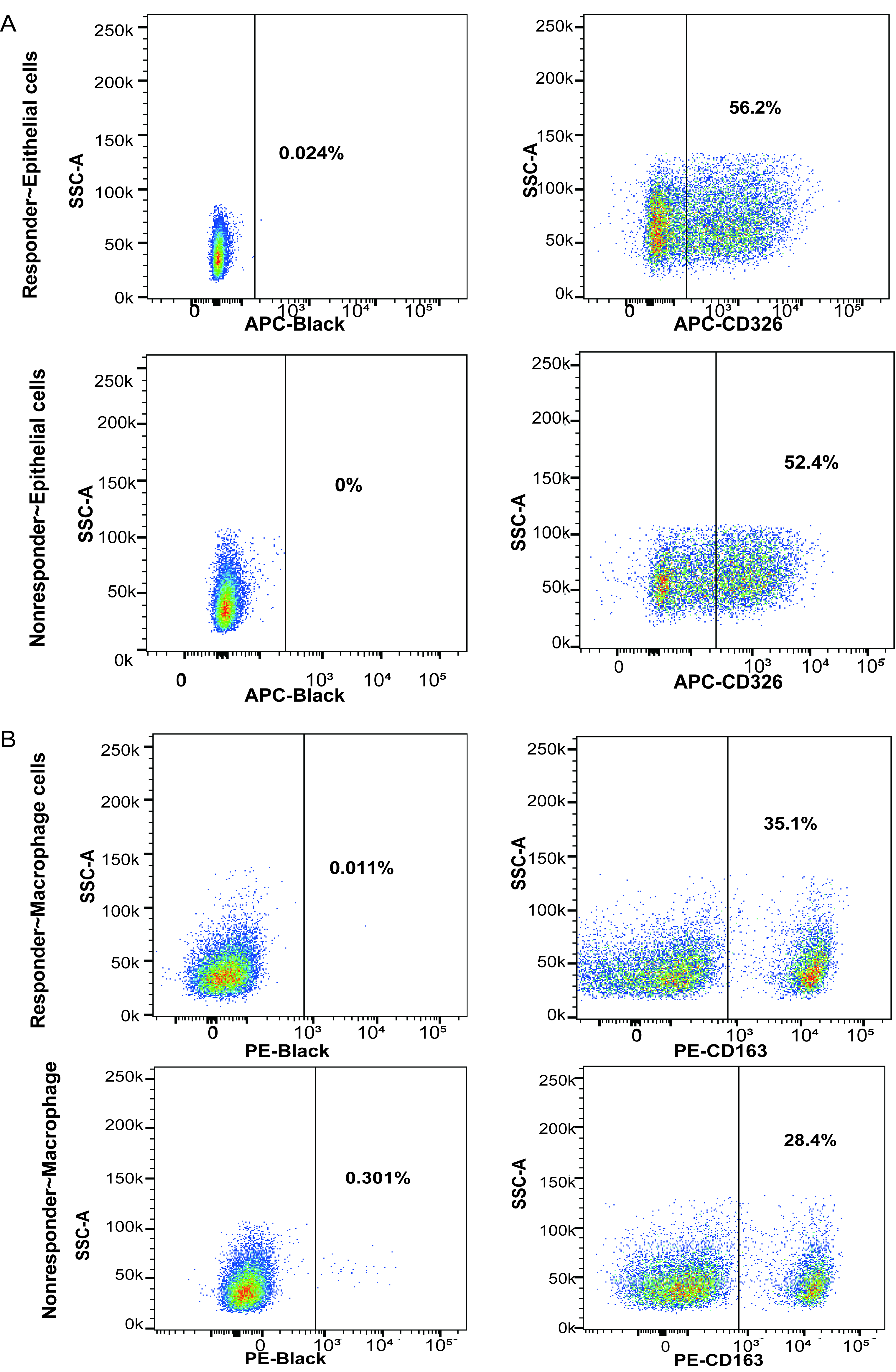

Supplement: Supplementary Figure 1 — Aberrant gene expression profiles in cell type-specific manners in LUAD with anti-PD-L1 treatment. (A) Volcano plot shows the differentially expressed genes (DEGs) of the tumor compared with the adjacent tissue analyzed by bulk RNA-seq datasets. (B) Malignant scores (x-axis) and nonmalignant scores are distributed on a scatter plot (y-axis). Color coding is used to represent density and assign each point to a cell. (C) Large-scale CNVs for each cell type are displayed on the heatmap. Tumor cells are represented in the bottom heatmap, and the expression levels for nonmalignant cells are plotted in the top heatmap, with genes arranged across the chromosomes from left to right. (D) Upregulated and downregulated gene scores were assessed in untreated patients, nonresponders, and responders with differentially expressed genes. (E) Pathway activities were scored for epithelial cells in LUAD patients using the “irGESA” algorithm. (F) Dotplot showing the relative expression of representative reported immune response genes and other representative genes in each cell type in nonresponder vs. untreated patients, responder vs. untreated patients, and responder vs. nonresponder. [file DataSheet_1.zip › FigureS2.tif]

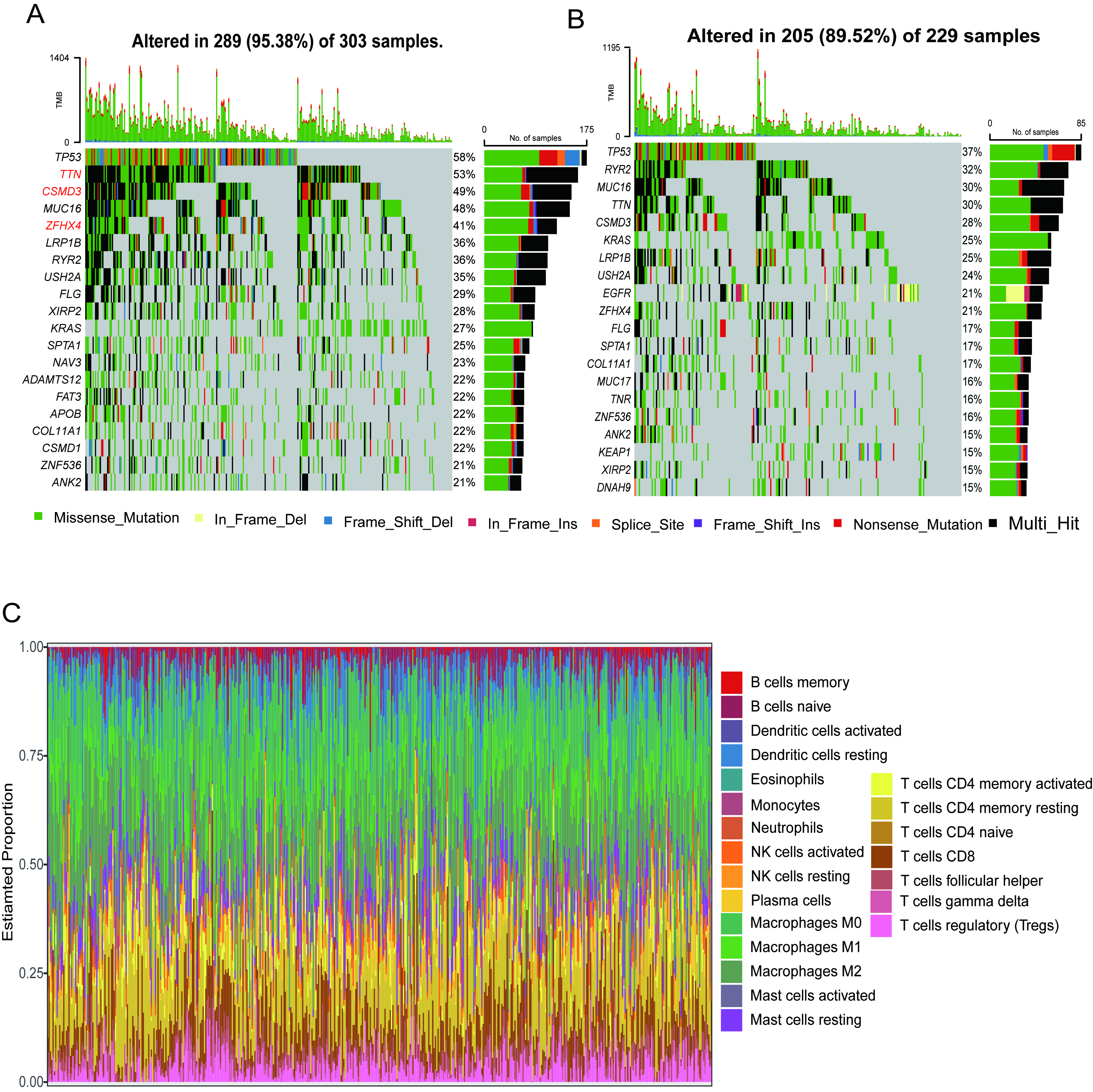

Supplement: Supplementary Figure 1 — Aberrant gene expression profiles in cell type-specific manners in LUAD with anti-PD-L1 treatment. (A) Volcano plot shows the differentially expressed genes (DEGs) of the tumor compared with the adjacent tissue analyzed by bulk RNA-seq datasets. (B) Malignant scores (x-axis) and nonmalignant scores are distributed on a scatter plot (y-axis). Color coding is used to represent density and assign each point to a cell. (C) Large-scale CNVs for each cell type are displayed on the heatmap. Tumor cells are represented in the bottom heatmap, and the expression levels for nonmalignant cells are plotted in the top heatmap, with genes arranged across the chromosomes from left to right. (D) Upregulated and downregulated gene scores were assessed in untreated patients, nonresponders, and responders with differentially expressed genes. (E) Pathway activities were scored for epithelial cells in LUAD patients using the “irGESA” algorithm. (F) Dotplot showing the relative expression of representative reported immune response genes and other representative genes in each cell type in nonresponder vs. untreated patients, responder vs. untreated patients, and responder vs. nonresponder. [file DataSheet_1.zip › FigureS3.tif]
